# Evaluation of Pharmacokinetics and Dose Proportionality of Diazepam After Intranasal Administration of NRL‐1 to Healthy Volunteers

**DOI:** 10.1002/cpdd.767

**Published:** 2020-01-09

**Authors:** Sarina Tanimoto, Luana Pesco Koplowitz, Richard E. Lowenthal, Barry Koplowitz, Adrian L. Rabinowicz, Enrique Carrazana

**Affiliations:** ^1^ Pacific Link Consulting San Diego California USA; ^2^ DUCK FLATS Pharma Elbridge New York USA; ^3^ Neurelis, Inc San Diego California USA

**Keywords:** NRL‐1, intranasal diazepam, pharmacokinetics, dose proportionality, seizure cluster, acute repetitive seizures

## Abstract

NRL‐1 is a novel intranasal formulation of diazepam that is being evaluated as rescue medication in patients with epilepsy who experience bouts of increased seizure activity despite stable regimens of antiepileptic drugs. This phase 1, open‐label, randomized, crossover study in healthy adult volunteers consisted of 3 single‐dose periods (5, 10, and 20 mg) followed by a 2‐dose period (2 × 10 mg) with a minimum 28‐day washout between treatments. Blood samples were taken at prespecified time points after intranasal dosing, and bioanalytic analysis of diazepam and nordiazepam was conducted using a validated liquid chromatography–tandem mass spectrometry method. Plasma pharmacokinetic parameters were summarized using descriptive statistics, and dose proportionality (peak concentration [C_max_] and area under the plasma concentration–time curve [AUC_0‐∞_]) was evaluated based on a power model within a 90%CI of 0.84 to 1.16. Comparisons were also conducted between single 10‐mg dose and multidose (2 × 10 mg) treatments. NRL‐1 administration resulted in rapid diazepam absorption (median time to peak concentration 1.4‐1.5 hours). Plasma concentration‐time profiles showed similar patterns of exposure that appeared to be dose dependent, with C_max_ of 85.6, 133.6, and 235.3 ng/mL for the 5‐, 10‐, and 20‐mg doses, respectively, although the lower 90%CI for C_max_ and AUC_0‐∞_ exceeded dose proportionality criteria. The coefficient of variation ranged from 59% to 67% for C_max_ and 48% to 56% for AUC parameters. Dose‐normalized AUC_0–∞_ values were comparable between the 2 × 10‐mg and single 10‐mg doses. Treatment‐emergent adverse events were consistent with those expected for diazepam, with transient somnolence the most frequent adverse event (94.4%). These results support NRL‐1 as a potential therapy for managing seizure emergencies.

Epilepsy affects approximately 50 million people worldwide,^1^ and the prevalence in the United States is almost 3.5 million individuals including 3 million adults and 470,000 children.^2^

Epilepsy is associated with a substantial disease burden that results from increased morbidity and mortality relative to the general population, its impact on patient and caregiver quality of life, and an increased need for healthcare resource use.^1^

Although management of epilepsy relies on the use of antiseizure drugs (ASD) to prevent and control seizures, up to 40% of individuals being treated with ASD may have refractory epilepsy.^3^ Bouts of increased seizure, often referred to as seizure clusters, are frequently experienced by some patients with epilepsy.^4^ These bouts can be defined as intermittent increases of seizure activity while on stable regimens of ASDs, and they require immediate rescue medication that can readily and easily be administered to manage these seizure emergencies. Rescue therapy has generally relied on use of benzodiazepines, and although intravenous administration is frequently used in an inpatient setting, rectal diazepam (Diastat) has been the standard of care in an outpatient setting and is appropriate for administration by non–healthcare professionals. However, because rectal administration may be difficult under seizure conditions and may be embarrassing to patients and caregivers, there is a need for easier and more socially acceptable routes of administration.

Intranasal administration can be considered an appropriate alternative with advantages that include being noninvasive and readily accessible to patients and caregivers, additionally providing a large absorptive surface area with high vascularity and direct nose to brain drug delivery, thus bypassing presystemic metabolism resulting from intestinal and hepatic first‐pass effects.^5^ Development of intranasal formulations of benzodiazepines has been problematic,^5^ mainly because of solubility and absorption issues related to their nonaqueous nature that have resulted in failure to achieve appropriate pharmacokinetic profiles in at least 1 prior intranasal formulation.^6^ However, recent advances have resulted in intranasal formulations of diazepam (NRL‐1; Valtoco) and midazolam nasal spray (USL261; Nayzilam). Although limited data have also suggested the efficacy of an intranasal formulation of lorazepam,^7^ the physicochemical properties of lorazepam are not as amenable as those of diazepam and midazolam to crossing the blood‐brain barrier through this route of administration^8^; lorazepam is less lipophilic than diazepam and midazolam.

Midazolam nasal spray has 62% to 73% absolute bioavailability and 125% to 149% relative bioavailability compared with intravenous midazolam in healthy adults, with a short time to maximum plasma concentration (t_max_; 10‐12 minutes) as well as short half‐life (t_½_; < 4 hours).^9^ In a clinical trial of intranasal midazolam, 53.7% of the midazolam‐treated patients met the primary efficacy endpoint (“treatment success,” defined as seizure termination within 10 minutes and no seizure recurrence at 6 hours) versus 34.3% with placebo (*P* < 0.05), and 58.2% of the midazolam‐treated patients remained seizure‐free at 6 hours after administration (compared with 37.3% of placebo‐treated patients).^10^ In a long‐term extension (median 16.8 months), 55% of seizure clusters met treatment success criteria after 1 dose.^11^ Midazolam nasal spray has received approval from the United States Food and Drug Administration for seizure clusters (acute repetitive seizures) in patients with epilepsy ≥12 years old.

In contrast to midazolam nasal spray, NRL‐1 is a unique intranasal preparation of diazepam that is formulated with vitamin E to increase diazepam's nonaqueous solubility and Intravail A3 (n‐dodecyl β‐D‐maltoside [DDM]), which is a nonionic surfactant that is used as an absorption enhancement agent to promote the increased transmucosal bioavailability of drugs.^12^ NRL‐1 is currently being investigated for intermittent use as rescue medication in patients with epilepsy who experience bouts of increased seizure activity despite stable regimens of antiepileptic drugs. NRL‐1 has less intrasubject pharmacokinetic variability than rectal diazepam, and although the t_max_ of intranasal diazepam is more than 1 hour, it has 97% bioavailability and a half‐life of ∼49 hours^13^; both the bioavailability and half‐life are higher relative to intranasal midazolam and may convey an advantage in reducing the need for a second rescue dose.

The purpose of the current study was to further assess the pharmacokinetics and dose proportionality of diazepam after intranasal administration of NRL‐1 to healthy volunteers.

## Methods

### Study Design

This phase 1, open‐label, randomized, crossover study consisted of 3 single‐dose periods encompassing 6 sequences followed by a 2‐dose period. The study was conducted at Novum Pharmaceutical Research Services (Houston, Texas) and received approval from an Institutional Review Board (Novum Independent, Pittsburgh, Pennsylvania) and was conducted in accordance with the Declaration of Helsinki; all subjects provided written informed consent before study participation.

The study was conducted on an inpatient basis at the study site, and subjects remained at the site for ∼36 hours in each treatment period, with a minimum 28‐day washout between treatments. NRL‐1 was administered as a 100‐µL intranasal spray of either 50 mg/mL or 100 mg/mL formulation under fasted conditions after an overnight fast. In the single‐dose periods, the 5‐ and 10‐mg doses were administered into a single nostril, and the 20‐mg dose required an administration in each nostril of the 100 mg/mL formulation. In the 2‐dose period, 2 10‐mg doses were given 4 hours apart in alternate nostrils.

### Subjects

Eligible subjects were healthy male and female adult volunteers 18 to 55 years old, inclusive, with a body weight 51 kg to 111 kg, inclusive. Subjects were also required to have no clinically significant abnormal findings in their medical history or on physical examination, electrocardiogram (QTcF < 450 msec for men and QTcF < 470 msec for women), or clinical laboratory results during screening. Female subjects of childbearing potential had to agree to use a medically acceptable method of contraception during the study duration, and those who were trying to conceive, were pregnant, or were lactating were excluded. Other key exclusion criteria were a history of major depression or a past suicide attempt or suicide ideation; a history of allergy or adverse response to diazepam; treatment with enzyme‐altering drugs in the past 30 days, over‐the‐counter oral and/or nasal decongestants in the past 14 days, or prescription medications, including benzodiazepines, in the past 14 days, excluding hormonal contraceptives.

### Bioanalytical Evaluation

Serial blood samples were collected into labeled K_2_‐EDTA tubes by direct venipuncture at prespecified time points. For the single‐dose treatment periods, collection times were at predose and at 10, 20, 30, and 45 minutes, and 1, 1.25, 1.5, 1.75, 2, 4, 8, 12, 24, 36, 48, 72, 96, 144, 192, and 240 hours after dosing. For the 2‐dose treatment period, blood was collected predose and at 10, 20, 30, and 45 minutes, and 1, 1.25, 1.5, 1.75, 2, 4, 4.167, 4.33, 4.5, 4.75, 5, 5.25, 5.5, 5.75, 6, 8, 12, 24, 36, 48, 72, 96, 144, 192, and 240 hours after the first dose. After collection, the plasma was separated by centrifugation (3000 rpm × 10 minutes at 0‐4°C at a relative centrifugal force of 2000g), and equal aliquots were transferred to 2 labeled tubes and stored at approximately –20°C until analysis.

Sample analysis was performed at a central laboratory (Quest Diagnostics, Houston, Texas) using a validated liquid chromatography–tandem mass spectrometry method to determine sample concentrations of diazepam and its metabolite nordiazepam (desmethyldiazepam) over the ranges of 1 to 1000 ng/mL and 100 to 100,000 pg/mL, respectively; 1.00 ng/mL and 100 pg/mL represent the lower limits of quantitation for diazepam and nordiazepam, respectively.

Analyte analysis consisted of automated liquid‐liquid extraction and separation using mobile phases of methanol/water with ammonium formate (mobile phase A) and methanol/water with acetic acid (mobile phase B). Separation was performed on an ACE 3 C18‐PFP column (30 × 4.6 mm, 3 µm; Advanced Chromatography Technologies Ltd, Aberdeen, Scotland) with detection using the API‐4000 system (Sciex, Framingham, Massachusetts).

Standard‐curve and quality control samples were generated by spiking blank human K_2_‐EDTA plasma (Bioreclamation; Westbury, New York) with internal standards (diazepam, nordiazepam, diazepam‐d_5_, and nordiazepam‐d_5_) obtained from Cerilliant (Round Rock, Texas) to monitor assay performance.

For precision, the intra‐assay results showed that the coefficient of variation (CV%) at the lower limit of quantitation was 4.0% for diazepam and 5.9% for nordiazepam; the corresponding intra‐assay accuracies, expressed as percentage bias, were 3.9% and –0.5% for diazepam and nordiazepam, respectively.

### Safety and Tolerability

Safety and tolerability were evaluated by the reporting of treatment‐emergent adverse events (TEAEs) regardless of causality, with additional determination as to whether the events were related to treatment. Evaluations also included clinical laboratory tests, physical examination, vital‐sign measurement, electrocardiography, clinical laboratory tests, and the Columbia Suicide Severity Rating Scale.^15^

To evaluate the potential for respiratory depression, oxygen saturation was measured using pulse oximetry at prespecified time points including within 1 hour predose, and postdose at 30 minutes and 1, 1.5, and 2 hours for the 20‐mg and first 10‐mg intranasal dose, and at 4.5, 5, 5.5, and 6 hours for the second 10‐mg intranasal dose. Nasal irritation and sedation were both measured objectively using 6‐point scales (0‐5, with higher scores indicative of greater severity) as previously described,^13^ with the former reported by a trained observer and the latter reported by the subject (if awake) and a trained observer. Changes in olfaction were assessed using the NIH Toolbox Odor Identification Test,^16^ and acute pain following nasal administration was assessed using a 0‐10 visual analogue scale (0 = no pain, 10 = extreme pain).

### Statistical and Pharmacokinetic Analyses

A target of 36 enrollees was planned with the goal of 24 completers. This sample size was determined to provide 97% power to conclude dose proportionality for C_max_ and AUC_0‐∞_ over the range of 5 mg to 20 mg based on 90%CI for the slope (β) from the power model^17^ within 0.84 and 1.16 with α = 0.05 and true slopes of 1. The analysis included a safety population, defined as subjects who received at least 1 dose of NRL‐1, and a pharmacokinetic population, defined as subjects who completed at least 2 of the 3 single doses or the 2 10‐mg treatments.

Pharmacokinetic parameters for diazepam and its metabolite, nordiazepam, were calculated from the individual plasma concentrations by noncompartmental methods using Phoenix WinNonlin version 7.0 (Certara Company, Princeton, New Jersey). The plasma pharmacokinetic parameters calculated were C_max_, t_max_, AUC_0‐t_, AUC_0‐∞_, t_½_, and apparent total body clearance (CL/F). For the 2‐dose period, C_max(0‐4h)_ and C_max(4‐8h)_ represent the maximum concentration within the 4 hours after the first dose and the maximum concentration in the 4–8‐hour time range, respectively, with similar representation of AUC_0‐4h_ and AUC_4‐8h_.

Plasma pharmacokinetic parameters were summarized using descriptive statistics. Analysis of variance was used to compare C_max_, AUC_0‐t_, and AUC_0‐∞_ among treatments with treatment, period, sequence, and subject within sequence as the classification variables using the natural logs. Comparisons were also conducted for C_max, 0‐4h_ and dose‐normalized AUC between the single‐dose (10 mg) and multidose (2 × 10 mg) treatments.

## Results

### Population

A total of 36 subjects were enrolled and received at least 1 dose of drug. Among the subjects who comprised the safety population, 69.4% were male, 75.0% were black (13.9% white and 11.1% other), the mean ± SD age was 36.0 ± 9.9 years, and mean ± SD body weight was 85.7 ± 13.4 kg. Twenty‐nine subjects completed the study, and of the 7 discontinuations, 6 (16.7%) were due to withdrawal by the subject and 1 (2.8%) was discontinued due to a protocol deviation (positive drug screen); none of the discontinuations was related to TEAEs. The pharmacokinetic population consisted of 33 patients.

### Pharmacokinetics

**Figure 1:**
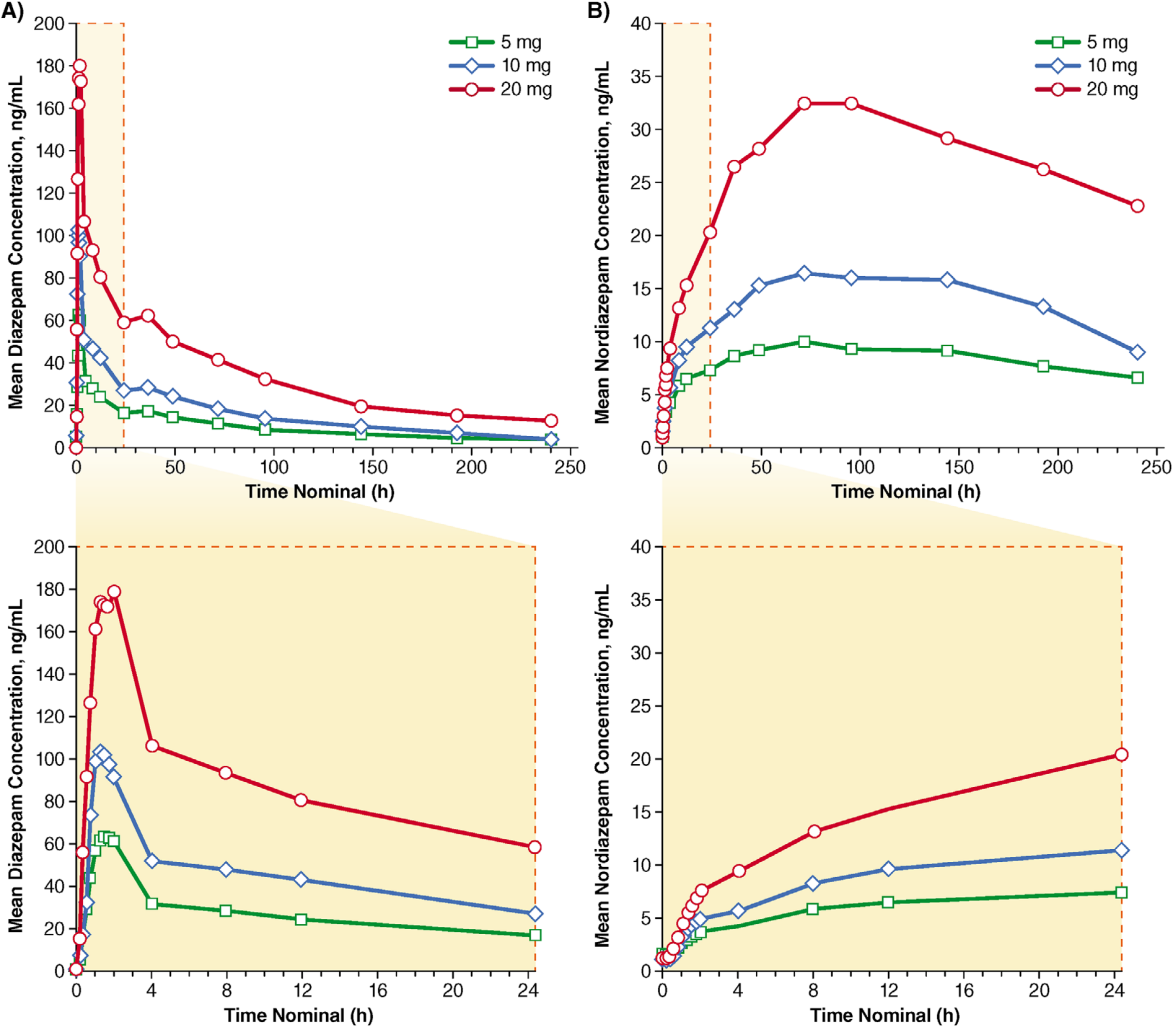
Arithmetic mean plasma concentration‐time profiles of diazepam (A) and nordiazepam (B) after single‐dose administration of NRL‐1.

Under single‐dose administration, there was rapid absorption of NRL‐1 across all doses (Table 1), with plasma concentration‐time profiles that showed similar patterns of exposure that appeared to be dose‐dependent (Figure 1A; Table 2). The arithmetic mean C_max_ values were 85.6, 133.6, and 235.3 ng/mL for the 5, 10, and 20 mg doses, respectively (Table 1). The arithmetic mean t_1/2_ and CL/F were comparable among the 3 doses (Table 1).

**Table 1:** Summary Statistics of Pharmacokinetic Parameters After Single Dose of NRL‐1

	Arithmetic Mean (CV%)			Geometric Mean (CV%)		
Pharmacokinetic Parameter	5 mg	10 mg	20 mg	5 mg	10 mg	20 mg
Diazepam						
C_max_, ng/mL	85.6 (67%)	133.6 (64%)	235.3 (59%)	64.2 (106%)	107 (82%)	199.6 (65%)
t_max_, h^a^	1.5 (0.3, 8)	1.5 (0.8, 36)	1.4 (0.5, 8)	…	…	…
AUC_0‐t_, h•ng/mL	2232 (51%)	3803 (52%)	7946 (52%)	1910 (68%)	3303 (62%)	6864 (63%)
AUC_0‐∞_, h•ng/mL	2411 (48%)	4505 (56%)	9168 (55%)	2110 (62%)	3854 (65%)	7711 (72%)
t_½_, h	70 (42%)	71 (44%)	74 (49%)	64 (47%)	65 (48%)	66 (49%)
CL/F, L/h	2.8 (72%)	3.1 (69%)	3.2 (75%)	2.4 (62%)	2.6 (65%)	2.6 (72%)
Nordiazepam						
C_max_, ng/mL	11.7 (48%)	19.3 (61%)	36.6 (57%)	10.5 (55%)	16.1 (71%)	31.6 (59%)
t_max_, h^a^	96 (8, 240)	96 (12, 240)	96 (36, 240)	…	…	…
AUC_0‐t_, h•ng/mL	1832 (46%)	3115 (55%)	6067 (53%)	1638 (54%)	2653 (66%)	5285 (59%)
AUC_0‐∞_, h•ng/mL	3229 (50%)	4503 (37%)	8838 (59%)	2972 (46%)	4258 (39%)	7462 (73%)
t_½_, h	91 (51%)	68 (29%)	85 (32%)	82 (52%)	66 (30%)	82 (28%)

AUC indicates area under the plasma concentration–time curve; AUC_0‐t_, AUC from time of dose to last measurement; AUC_0–∞_, AUC from time of dose extrapolated to infinity; C_max_, peak plasma concentration; CV%, coefficient of variation; t_max_, time from dose to C_max_; t_½_, half‐time of elimination.

a Values are medians (minimum, maximum). Ranges in subscripts indicate time intervals of measurements.

**Table 2:** Summary Statistics of Pharmacokinetic Parameters After Multiple Dose (2 × 10 mg) of NRL‐1

Pharmacokinetic Parameter	Arithmetic Mean (CV%)	Geometric Mean (CV%)
Diazepam		
C_max,0‐4h_, ng/mL	123.0 (73%)	91.5 (100%)
C_max,4‐8h_, ng/mL	161.4 (77%)	114.6 (113%)
C_max_, ng/mL	180.2 (68%)	137.2 (97%)
t_max_, h^a^	5.3 (0.5, 12)	…
AUC_0‐4h_, h•ng/mL	282 (71%)	212 (98%)
AUC_4‐8h,_ h•ng/mL	433 (70%)	327 (97%)
AUC_0‐t_, h•ng/mL	7297 (58%)	6067 (73%)
AUC_0‐∞_, h•ng/mL	7936 (57%)	6831 (61%)
t_½_, h	74 (45%)	67 (50%)
CL/F, L/h	3.4 (59%)	2.9 (61%)
Nordiazepam		
C_max,0‐4h_, ng/mL	6.6 (71%)	5.2 (81%)
C_max,4‐8h_, ng/mL	11.7 (78%)	9.1 (82%)
C_max_, ng/mL	32.1 (55%)	26.7 (75%)
t_max_, h^a^	144 (24, 240)	…
AUC_0‐4h_, h•ng/mL	16 (64%)	13 (68%)
AUC_4‐8h_ h•ng/mL	38 (77%)	29 (83%)
AUC_0‐t_, h•ng/mL	5625 (51%)	4796 (68%)
AUC_0‐∞_, h•ng/mL	10 840 (23%)	10 595 (26%)
t_½_, h	69 (21%)	67 (22%)

AUC indicates area under the plasma concentration–time curve; C_max_, peak plasma concentration; CV%, coefficient of variation; t_max_, time from dose to C_max_; t_½_, half‐time of elimination.

a Values are medians (minimum, maximum). Ranges in subscripts indicate time intervals of measurements.

The plasma concentration‐time profiles for nordiazepam generally showed similar patterns to those of diazepam across the 3 NRL‐1 doses (Figure 1B), with identical median t_max_ values of 96 hours. Nordiazepam C_max_ appeared to be dose‐dependent (Table 1), although there was substantial interpatient variability, which ranged from 48% to 61% across the doses, and t_½_ was lowest after 10‐mg administration.

**Figure 2:**
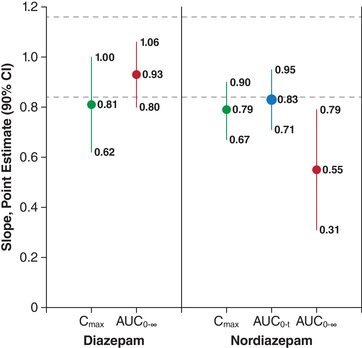
Analysis of dose proportionality. Broken lines indicate the 90%CI criteria (0.84, 1.16) for meeting dose proportionality. AUC indicates area under the plasma concentration–time curve; AUC_0‐t_, AUC from time of dose to last measurement; AUC_0–∞_, AUC from time of dose extrapolated to infinity; C_max_, peak plasma concentration.

In the dose proportionality assessment for diazepam, the slope estimates were 0.81 (90%CI 0.62, 1.00) for C_max_ and 0.93 (90%CI 0.80, 1.06) for AUC_0‐∞_. Both of the 90%CIs exceeded the formal dose proportionality criteria on the lower limit (Figure 2). The 90%CIs were also exceeded for the evaluated parameters of nordiazepam (C_max_, AUC_0‐t_, and AUC_0‐∞_; Figure 2).

The pharmacokinetic parameters observed during the multiple‐dose phase are summarized in Table 2. Under conditions of multiple dosing, the diazepam geometric mean C_max_ value after the second dose (C_max,4‐8h_, 114.6 ng/mL; CV% 113%) was approximately 1.25 times higher than that after the first dose (C_max,0‐4h_, 91.5 ng/mL; CV% 100%). Similarly, the diazepam geometric mean for drug exposure was 1.54 times higher after the second dose (AUC_4‐8h_) relative to that of the first dose (AUC_0‐4h_), 327 h•ng/mL (CV% 97%) and 212 h•ng/mL (CV%, 98%), respectively. In the case of nordiazepam, the C_max_ and AUC geometric means for the second dose were, respectively, approximately 1.75 and 2.19 times higher than those for the first dose.

**Figure 3:**
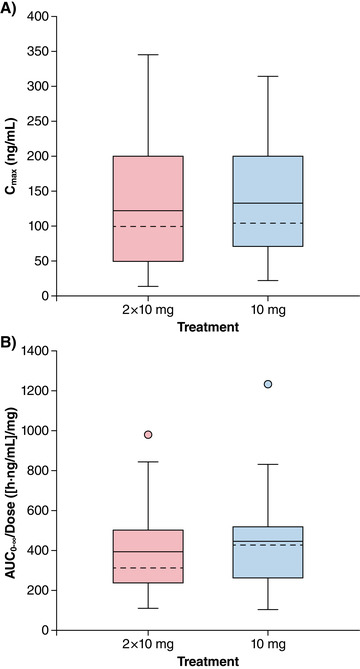
Box‐and‐whisker plots of diazepam C_max,0‐4h_ multiple‐dose and C_max_ of single‐dose diazepam (A) and dose‐normalized AUC_0–∞_ multiple‐ and single‐dose (B) after intranasal administration of NRL‐1. The dashed line (‐ ‐ ‐) is the median; the solid line (–) is the arithmetic mean. The ends of the “box” (⊥) are the first and third quartiles, and the whiskers show the lowest and highest data values still within 1.5 of the interquartile range of the lower and upper quartiles, respectively. Data values that do not fall between the whiskers are plotted as markers outside of the whiskers.

Comparison of the 2 × 10‐mg dose with the single 10‐mg dose using box plots showed that values for C_max,0‐4h_ were similar to that of the single‐dose 10 mg C_max_ (Figure 3A). The dose‐normalized AUC_0–∞_ values were also comparable between the 2 × 10‐mg and single 10‐mg doses (Figure 3B).

### Safety and Tolerability

Nearly all the subjects (97.2%) had at least 1 TEAE, and the TEAEs were considered treatment‐related in 94.4% of the subjects (Table 3). There were no serious TEAEs and no discontinuations due to TEAEs. Somnolence was the most frequent TEAE (94.4%) and had the highest incidence with multiple dosing (61.1%). Most of the other common TEAEs occurred only with multiple dosing (Table 3).

**Table 3:** Incidence of Treatment‐Emergent Adverse Events in the Safety Population (N = 36)

	Incidence, n (%)				
TEAE	NRL‐1 5 mg	NRL‐1 10 mg	NRL‐1 20 mg	NRL‐1 2 × 10 mg	Total
Any TEAE	17 (47.2)	13 (36.1)	19 (52.8)	24 (66.7)	35 (97.2)
Serious TEAEs	0	0	0	0	0
Discontinuations due to TEAEs	0	0	0	0	0
Treatment‐related TEAEs	16 (44.4)	13 (36.1)	19 (52.8)	22 (61.1)	34 (94.4)
Most common TEAEs (≥5% at any dose)					
Somnolence	16 (44.4)	13 (36.1)	18 (50.0)	22 (61.1)	34 (94.4)
Reticulocyte count increased	0	0	0	4 (11.1)	4 (11.1)
Euphoric mood	0	1 (2.8)	2 (5.6)	0	3 (8.3)
Blood glucose increased	0	0	0	3 (8.3)	3 (8.3)
Hemoglobin decreased	0	0	0	3 (8.3)	3 (8.3)
Urine analysis abnormal	0	0	0	3 (8.3)	3 (8.3)
Alanine aminotransferase increased	0	0	0	2 (5.6)	2 (5.6)
Blood pressure decreased	0	0	2 (5.6)	0	2 (5.6)

TEAE indicates treatment‐emergent adverse event.

There were no reports of pain on the pain visual analogue scale after administration of NRL‐1, and nasal pain was not reported as a TEAE. No dose‐ or time‐related trends were observed in nasal irritation, and the majority of subjects (65.5%‐100%) had no nasal irritation (data not shown). Nearly all of the nasal irritation that was observed was rated as a score of 1 (“inflamed mucosa, no bleeding”), and the highest score was 2 (“minor bleeding which stops within 1 minute”), which was observed in the same subject at 1 and 2 hours after administration of the 5‐mg dose. Changes in olfaction were generally small and did not appear to be dose dependent (data not shown).

There were no trends in sedation (data not shown), and all but 1 of the sedation scores were 2 (“napping or sleeping, but easily awakened”) or less; the 1 exception was a subject who had a score of 3 (“sleeping, awakened only with loud voice or shaking”) at 2 hours after administration of a 20‐mg dose.

Blood oxygen saturation levels postdose were consistently within normal limits (94% to 100%). Suicide ideation and behavior were reported on the Columbia Suicide Severity Rating Scale by a single subject. This 1 subject, who reported suicidal ideation but no suicidal behavior at baseline and was subsequently enrolled in the study, reported suicidal ideation and behavior postdose on day 0 after administration of 10 mg but on no other study days.

## Discussion

The use of DDM and vitamin E has resolved the issues related to the nonaqueous nature of diazepam by enhancing both solubility and absorption of diazepam; NRL‐1 is the only nonaqueous drug formulation that uses DDM. As shown by the pharmacokinetic results, administration of NRL‐1 in healthy adult volunteers resulted in rapid absorption of diazepam, with a C_max_ that was reached approximately 1.5 hours after a nominal lag time. Diazepam C_max_ after intranasal administration of NRL‐1 was lower than what may be expected based on the pharmacokinetics of intravenous (95‐125 ng/mL) and oral (148‐255 ng/mL) administration.^18^ However, C_max_ and exposure (AUC) were generally consistent with the range that has been reported with rectal diazepam.^19^, ^20^, ^21^, ^22^, ^23^

Systemic exposure increased with higher doses. However, formal testing of dose proportionality was inconclusive due to the intersubject variability that is a well‐recognized characteristic of the pharmacokinetics of diazepam.^13^, ^19^, ^20^, ^21^, ^22^, ^24^, ^25^ Despite this variability, the observed exposure over the dose range suggests that there is likely to be a predictable and consistent dose‐response relationship. With further regard to the variability of NRL‐1, it should be noted that the CV% of the geometric mean C_max_ and AUC in the current study, which included subjects over a wide weight range, was consistent with what has been previously reported in a study that compared NRL‐1 with rectal diazepam administered to healthy volunteers under “ideal” conditions (ie, fasting state with an enema the night prior to dosing and a second enema 1 hour prior to dosing the following morning).^23^ In that study, NRL‐1 demonstrated less variability than rectal diazepam, which was characterized by pharmacokinetics that appeared to be weight dependent, with substantially higher CV% among heavier subjects. Taken together, these results suggest that, regardless of weight, NRL‐1 provides more reliable pharmacokinetics than rectal administration.

In the double‐dose period of the current study, the observed absorption and exposure indicated that it is easy to increase the dose as needed. Multiple doses of NRL‐1 (2 × 10 mg separated by 4 hours) resulted in an initial diazepam C_max,0‐4h_ and AUC_0‐4h_ comparable to that observed for the single 10‐mg dose. Dose‐normalized AUCs were similar between the single and multiple 10‐mg doses. The second dose (administered 4 hours following the first dose) resulted in accumulation in diazepam C_max_ and AUC 4 hours postdose. These observations are in contrast to concentration effects that often drive exposure with dermal and transmembrane administration.^26^, ^27^, ^28^ It should also be noted that the approximately 2‐fold higher C_max_ and AUC values of nordiazepam after the second dose relative to the first dose were not reflective of accumulation, as the consistent rising of nordiazepam concentrations was delayed beyond the 8‐hour postinitial dose. However, it should be noted that nordiazepam is an active metabolite, and despite the report that the absolute extent of conversion from diazepam is only about 55%,^29^ the long half‐lives of both diazepam and its metabolite could potentially result in accumulation with frequent dosing, as suggested by a study of oral diazepam pharmacokinetics in obese individuals.^14^ Although that study showed greater accumulation of both diazepam and nordiazepam under conditions of chronic use among obese individuals, subjects were dosed daily for 1 month, a dosing frequency that would not be generally expected when used as a rescue medication; NRL‐1 is an intermittent treatment for seizure clusters that is unlikely to create accumulation.

Although NRL‐1 has not been evaluated in humans in the absence of DDM, it may be expected that the favorable pharmacokinetic characteristics that support this intranasal formulation are derived from the increased solubility and drug absorption associated with the proposed mechanism of DDM. This mechanism is based on enhanced absorption via relaxation of tight junctions combined with fluidization and greater penetration through cell membranes.^12^

Overall, NRL‐1 demonstrated a good safety profile in these healthy adult volunteers, with TEAEs that were consistent with what may be expected for diazepam. Although somnolence was the main TEAE, only low levels of transient sedation were reported, and no evidence for respiratory depression was observed. Furthermore, there were no clinically relevant changes in any of the clinical/laboratory tests. Administration of NRL‐1 demonstrated good tolerability, with no reports of pain and only minimal nasal irritation and changes in olfaction that were not clinically relevant.

This study was performed in healthy adult volunteers, and the results cannot be directly extrapolated to patients with epilepsy and to pediatric patients in particular. However, preliminary evidence from a recent phase 1 study in patients with epilepsy suggests that the pharmacokinetics and safety of NRL‐1 administered to children and adolescents under ictal/peri‐ictal, and nonictal conditions are similar to those in adults.^30^

## Conclusions

We may conclude there to be good absorption and exposure of diazepam with intranasal administration of NRL‐1 across the range of dosing; exposure with increasing dose was indicative of dose proportionality. NRL‐1 was also associated with overall safety and tolerability consistent with what is known for diazepam, which has been well characterized. These results further support NRL‐1 as a potential novel therapeutic approach to the management of seizure emergencies using a formulation that provides easy and socially acceptable administration during the intermittent occurrence of seizure clusters.

## Conflicts of Interest

S.T. is an employee of Pacific Link Consulting Services, which provided consulting services to Neurelis, and has a leadership position with ARS Pharmaceuticals, Inc, which has an agreement with Neurelis for the use of Intravail. L.P.K. is an officer of DUCK FLATS Pharma, which provided consulting services to Neurelis. R.E.L. is an employee of Pacific Link Consulting Services, which provided consulting services to Neurelis, and has a leadership position with ARS Pharmaceuticals, Inc, which has an agreement with Neurelis for the use of Intravail. B.K. is an employee of DUCK FLATS Pharma, which provided consulting services to Neurelis. A.L.R. is an employee and has received stock options from Neurelis. E.C. has received personal compensation for consulting with Neurelis, Alexza, Marinus, and Zogenix and holds stock in Neurelis. E.C. has received compensation for serving on the Board of Directors of Marinus Pharmaceuticals, Epalex, and Hawaii‐Biotech.

## Funding

This study was supported by Neurelis, Inc.

## Data‐Sharing Statement

Neurelis, Inc will not be sharing individual deidentified participant data or other relevant study documents.

## References

[1] G. B. D. Epilepsy Collaborators . Global, regional, and national burden of epilepsy, 1990‐2016: a systematic analysis for the Global Burden of Disease Study 2016. Lancet Neurol. 2019;18(4):357‐375. 30773428 10.1016/S1474-4422(18)30454-X PMC6416168

[2] Centers for Disease Control and Prevention . Epilepsy Data and Statistics. https://www.cdc.gov/epilepsy/data/index.html. Accessed April 22, 2019.

[3] Laxer KD , Trinka E , Hirsch LJ , et al. The consequences of refractory epilepsy and its treatment. Epilepsy Behav. 2014;37:59‐70. 24980390 10.1016/j.yebeh.2014.05.031

[4] Jafarpour S , Hirsch LJ , Gainza‐Lein M , Kellinghaus C , Detyniecki K . Seizure cluster: definition, prevalence, consequences, and management. Seizure. 2019;68:9‐15. 29871784 10.1016/j.seizure.2018.05.013

[5] Kapoor M , Cloyd JC , Siegel RA . A review of intranasal formulations for the treatment of seizure emergencies. J Control Release. 2016;237:147‐159. 27397490 10.1016/j.jconrel.2016.07.001

[6] Acorda Therapeutics . PLUMIAZ™ (diazepam) Nasal Spray [press release]. http://www.acorda.com/products/research-development/plumiaz. Accessed July 1, 2019.

[7] Allan A , Cullen J . Best BETs from the Manchester Royal Infirmary. BET 1: intranasal lorazepam is an acceptable alternative to intravenous lorazepam in the control of acute seizures in children. Emerg Med J. 2013;30(9):768‐769. 23943640 10.1136/emermed-2013-202981.1

[8] Maglalang PD , Rautiola D , Siegel RA , et al. Rescue therapies for seizure emergencies: new modes of administration. Epilepsia. 2018;59 Suppl 2:207‐215. 30159892 10.1111/epi.14479

[9] Bancke LL , Dworak HA , Rodvold KA , Halvorsen MB , Gidal BE . Pharmacokinetics, pharmacodynamics, and safety of USL261, a midazolam formulation optimized for intranasal delivery, in a randomized study with healthy volunteers. Epilepsia. 2015;56(11):1723‐1731. 26332539 10.1111/epi.13131

[10] Detyniecki K , Van Ess PJ , Sequeira DJ , Wheless JW , Meng TC , Pullman WE . Safety and efficacy of midazolam nasal spray in the outpatient treatment of patients with seizure clusters—a randomized, double‐blind, placebo‐controlled trial. Epilepsia. 2019;60(9):1797‐1808. 31140596 10.1111/epi.15159 PMC9291143

[11] Wheless JW , Meng TC , Van Ess PJ , Detyniecki K , Sequeira DJ , Pullman WE . Safety and efficacy of midazolam nasal spray in the outpatient treatment of patients with seizure clusters: An open‐label extension trial. Epilepsia. 2019;60(9):1809‐1819. 31353457 10.1111/epi.16300

[12] Maggio ET , Pillion DJ . High efficiency intranasal drug delivery using Intravail^®^ alkylsaccharide absorption enhancers. Drug Deliv Transl Res. 2013;3(1):16‐25. 25787864 10.1007/s13346-012-0069-z

[13] Agarwal SK , Kriel RL , Brundage RC , Ivaturi VD , Cloyd JC . A pilot study assessing the bioavailability and pharmacokinetics of diazepam after intranasal and intravenous administration in healthy volunteers. Epilepsy Res. 2013;105(3):362‐367. 23561287 10.1016/j.eplepsyres.2013.02.018

[14] Abernethy DR , Greenblatt DJ , Divoll M , Shader RI . Prolonged accumulation of diazepam in obesity. J Clin Pharmacol. 1983;23(8‐9):369‐376. 6415130 10.1002/j.1552-4604.1983.tb02750.x

[15] Posner K , Brown GK , Stanley B , et al. The Columbia‐Suicide Severity Rating Scale: initial validity and internal consistency findings from three multisite studies with adolescents and adults. Am J Psychiatry. 2011;168(12):1266‐1277. 22193671 10.1176/appi.ajp.2011.10111704 PMC3893686

[16] Dalton P , Doty RL , Murphy C , et al. Olfactory assessment using the NIH Toolbox. Neurology. 2013;80(11 Suppl 3):S32‐S36. 23479541 10.1212/WNL.0b013e3182872eb4 PMC3662337

[17] Sethuraman VS , Leonov S , Squassante L , Mitchell TR , Hale MD . Sample size calculation for the power model for dose proportionality studies. Pharm Stat. 2007(6):1. 17323313 10.1002/pst.241

[18] Divoll M , Greenblatt DJ , Ochs HR , Shader RI . Absolute bioavailability of oral and intramuscular diazepam: effects of age and sex. Anesth Analg. 1983;62(1):1‐8. 6849499

[19] Cloyd JC , Lalonde RL , Beniak TE , Novack GD . A single‐blind, crossover comparison of the pharmacokinetics and cognitive effects of a new diazepam rectal gel with intravenous diazepam. Epilepsia. 1998;39(5):520‐526. 9596205 10.1111/j.1528-1157.1998.tb01415.x

[20] Henney HR 3rd , Sperling MR , Rabinowicz AL , Bream G , Carrazana EJ . Assessment of pharmacokinetics and tolerability of intranasal diazepam relative to rectal gel in healthy adults. Epilepsy Res. 2014;108(7):1204‐1211. 24934774 10.1016/j.eplepsyres.2014.04.007

[21] Lamson MJ , Sitki‐Green D , Wannarka GL , Mesa M , Andrews P , Pellock J . Pharmacokinetics of diazepam administered intramuscularly by autoinjector versus rectal gel in healthy subjects: a phase I, randomized, open‐label, single‐dose, crossover, single‐centre study. Clin Drug Invest. 2011;31(8):585‐597. 10.2165/11590250-000000000-00000 21721594

[22] Ivaturi V , Kriel R , Brundage R , Loewen G , Mansbach H , Cloyd J . Bioavailability of intranasal vs. rectal diazepam. Epilepsy Res. 2013;103(2‐3):254‐261. 22981338 10.1016/j.eplepsyres.2012.07.018

[23] Hogan RE , Gidal BE , Koplowitz B , Koplowitz LP , Lowenthal RE , Carrazana E . Bioavailability and safety of Valtoco™ (diazepam intranasal solution) compared to oral and rectal diazepam. Poster presented at the 72nd Annual Meeting of the American Epilepsy Society, November 30–December 4, 2018, New Orleans, LA.

[24] Ivaturi VD , Riss JR , Kriel RL , Cloyd JC . Pharmacokinetics and tolerability of intranasal diazepam and midazolam in healthy adult volunteers. Acta Neurol Scand. 2009;120(5):353‐357. 19456308 10.1111/j.1600-0404.2009.01170.x

[25] Ivaturi VD , Riss JR , Kriel RL , Siegel RA , Cloyd JC . Bioavailability and tolerability of intranasal diazepam in healthy adult volunteers. Epilepsy Res. 2009;84(2‐3):120‐126. 19231138 10.1016/j.eplepsyres.2009.01.001

[26] Schneider T , Vermeulen R , Brouwer DH , Cherrie JW , Kromhout H , Fogh CL . Conceptual model for assessment of dermal exposure. Occup Environ Med. 1999;56(11):765‐773. 10658563 10.1136/oem.56.11.765 PMC1757678

[27] Tregear RT . Physical functions of skin. London, New York: Academic Press; 1966.

[28] Wester RC , Maibach HI . Relationship of topical dose and percutaneous absorption in rhesus monkey and man. J Invest Dermatol. 1976;67(4):518‐520. 823268 10.1111/1523-1747.ep12664543

[29] Greenblatt DJ , Divoll MK , Soong MH , Boxenbaum HG , Harmatz JS , Shader RI . Desmethyldiazepam pharmacokinetics: studies following intravenous and oral desmethyldiazepam, oral clorazepate, and intravenous diazepam. J Clin Pharmacol. 1988;28(9):853‐859. 2906643 10.1002/j.1552-4604.1988.tb03228.x

[30] Tarquinio D , Segal E , Wheless JW , Rabinowicz AL , Carrazana E , the DIAZ 001.04 Study Group . Pharmacokinetics and safety of Valtoco™ (NRL‐1; diazepam nasal spray) in children with epilepsy during seizure (ictal/peri‐ictal) and non‐seizure (inter‐ictal) conditions: results from a phase 1, open‐label study. Poster presented at the 48th Annual Meeting of the Child Neurology Society, October 23‐26, 2019, Charlotte, NC.

